# Case Series of Endoscopic Papillary Balloon Dilation for Children with Common Bile Duct Stones and a Review of the Literature

**DOI:** 10.3390/jcm13082251

**Published:** 2024-04-12

**Authors:** Katsunori Kouchi, Ayako Takenouchi, Aki Matsuoka, Kiyoaki Yabe, Hiroko Yoshizawa, Chikako Nakata, Jun Fujishiro, Harutoshi Sugiyama

**Affiliations:** 1 Department of Pediatric Surgery, Tokyo Women’s Medical University, Ohwadashinden 477-96, Yachiyo 276-8524, Japan; ayako-t@gd5.so-net.ne.jp (A.T.); akimatsuoka13@gmail.com (A.M.); akiyb17@gmail.com (K.Y.); hyoshizawa1357@gmail.com (H.Y.);; 2 Department of Pediatric Surgery, Tokyo University, Hongou 7-3-1, Bunkyou-ku, Tokyo 113-8655, Japan; fujishiroj-psu@h.u-tokyo.ac.jp; 3 Department of Gastroenterology, Tokyo Women’s Medical University, 8-1 Shinjuku-ku, Tokyo 162-8666, Japan; sugiyama.harutoshi@twmu.ac.jp

**Keywords:** endoscopic papillary balloon dilation, common bile duct stone, choledocholithiasis, children, endoscopic retrograde cholangiopancreatography, endoscopic sphincterotomy

## Abstract

**Background**: Endoscopic sphincterotomy (EST) has been employed for the endoscopic treatment of common bile duct stones (CBDSs) and has been reported to have a high success rate for stone removal. However, EST is associated with a risk of bleeding, perforation, and sphincter of Oddi function disruption. To avoid these risks, endoscopic papillary balloon dilation (EPBD) is an option for CBDS. Sphincter of Oddi function preservation decreases long-term biliary infection and gallstone recurrence. EPBD may have advantages in children who require a long follow up. However, there have been few reports on pediatric cases, particularly in infants. **Methods**: From September 2017 to December 2023, we performed EPBD for four pediatric CBDSs. The patients were aged from 5 months to 8 years, including two infants aged 5 and 6 months. Furthermore, we reviewed the stone removal rate and complications of 545 ESTs performed at high-volume centers and 13 EPBD-reported cases in children with CBDSs. **Results**: CBDSs of all patients who underwent EPBD in our institution were successfully removed. No bleeding or perforation was noted; pancreatitis was observed in three patients. In an analysis of 545 ESTs in children, the stone removal rate was high, ranging from 83% to 100% (mean 96%). The incidence of pancreatitis was 0–9.6% (mean 4.4%), and the grade of pancreatitis was almost mild. The bleeding frequency was 1.3–5.4% (mean 2.7%). With regards to the grade of bleeding, seven cases were mild (64%) and four were moderate (36%). Compared with adults who underwent EST, the frequencies of pancreatitis and bleeding were almost equal in children; however, in children, once bleeding occurs, it has a higher risk of leading to blood transfusion. Stone removal via EPBD in children has a 100% success rate. Pancreatitis was responsible for all complications were related; its frequency was 46% (6/13 patients, including five mild cases and one moderate case), which is higher than that of EST and adult cases who underwent EPBD. In most children with pancreatitis, pancreatic enzyme levels returned to normal within 2–3 days following EPBD, and no severe cases caused by EPBD were reported. **Conclusions**: CBDS removal via EPBD in children has a high success rate with very low risk of bleeding and perforation. Although pancreatitis frequently occurs, most cases are mild. Sphincter of Oddi function preservation via EPBD is expected to prevent long-term stone recurrence and biliary tract infection, and EPBD is considered to be an effective method for CBDS removal in children.

## 1. Introduction

Gallbladder stones (GBSs) in children are less common than in adults, with a reported prevalence of 0.13–0.2% in Italy [1], 0.13% in Japan [2], and 1.9% in the Netherlands [3]. Recently, the prevalence of GBS has increased from 1.9% to 4% in children [4]. In infants, the most common causes of cholelithiasis are preterm birth, total parenteral nutrition, and abdominal surgery, whereas in children, the main causes are hemolytic disease and hereditary erythrocytosis [4,5]. Obesity and oral contraceptive use have recently been identified as risk factors in adolescents [5]. More than 80% of adults with GBSs are asymptomatic [6]. In contrast, 17–50% of children are symptomatic [6,7,8,9]. Common bile duct stones (CBDSs) require urgent treatment. Surgical treatment is invasive. Pogoretic et al. [10] proposed laparoscopic exploration of the common bile duct (LCBDE) as an alternative method; this has the advantage of simultaneous cholecystectomy and CBDS removal under laparoscopy using a flexible choledochoscope. In this series, children with CBDSs had a mean age and body weight of 11.4 years and 55.9 kg, respectively. As the diameter of the choledochoscope is 3.1–4 mm, we believe that LCBDE is not suitable for narrow common bile ducts or infants and is therefore mainly considered for older children. Endoscopic sphincterotomy (EST) has been employed to remove CBDSs in children [11,12,13,14,15,16,17,18]. To the best of our knowledge, a 3-month-old boy who developed CBDSs following chemotherapy for a malignant tumor is the youngest reported case of stone removal via EST [19]. As EST involves an incision of the main papilla, there is a risk of perforation and bleeding. Moreover, sphincter of Oddi function disruption could cause bacterial invasion and growth in the bile ducts and reflux of digestive juices [20,21,22], which are believed to be factors in bile duct stone recurrence [23,24,25].

In contrast, endoscopic papillary balloon dilation (EPBD) [26] has a lower risk of bleeding and perforation [24,25]; in addition, it preserves sphincter of Oddi function [27,28]. In children, sphincter of Oddi function preservation is desirable, owing to the long clinical course required. Therefore, although we previously performed EST for CBDSs, since September 2017 we have selected EPBD in order to preserve the sphincter of Oddi function. Only a few studies of EPBD for pediatric CBDSs have been conducted [29,30,31], especially in infants. In this study, we performed EPBD for stone removal in four children with CBDSs, two of whom were infants. We report our experience with a review of the literature on EPBD and EST in children with CBDSs.

## 2. Materials and Methods

### 2.1. Patients

From September 2017 to December 2023, four pediatric CBDSs requiring endoscopic treatment presented to our institution. Their ages ranged from 5 months to 8 years, two of whom were infants aged 5 and 6 months. A 5-month-old girl had no specific medical history and a 6-month-old girl had undergone open heart surgery for cardiac disease. The other two cases were 7- and 8-year-old boys. The CBDSs were 3–4 mm in size and 1–2 in number. The details of these cases are presented in Table 1.

### 2.2. Methods

Endoscopic retrograde cholangiopancreatography (ERCP) was performed under general anesthesia using PJF 240 (Olympus, Tokyo, Japan) in infants and TJFQ180 or JF260v (Olympus) in older children. The PJF 240 uses a video system and has a narrow tip diameter of 8.8 mm, making it useful for ERCP in infants. For older children, a balloon catheter with a 4–6 mm diameter and a 30 mm long balloon was used (ZARA; Century Medical Inc., Tokyo, Japan). The 30 mm long balloon is sufficiently long to be used for EPBD in infants. Shorter endoscopic papillary balloons are not available in the market. Therefore, we used a percutaneous transluminal coronary angioplasty (PCTA) balloon with the consent of the family for use in infants. The PCTA balloon is available in short balloon lengths. For infants, a catheter with a 3–4 mm diameter and a 15 mm long balloon (Ryurei; Terumo Inc., Tokyo, Japan) was used. The balloon was inflated for 30 s or 1 min. Following balloon dilatation, the balloon was deflated and inserted into the hepatic duct. To remove the bile stones, the balloon was subsequently reinflated and pulled back into the duodenum. Basket forceps and mechanical lithotripsy tool were not used. Following EPBD, a 4- or 5-Fr endoscopic nasobiliary drainage (ENBD) tube was prophylactically inserted in all patients. In our series, none of the children had received other prophylaxis for pancreatitis (hyper-hydration or indomethacin suppositories). The grading of endoscopic complications (pancreatitis and hemorrhage) [32] was classified according to Cotton et al. [33]. The Revised Atlanta classification of post-ERCP pancreatitis (PEP) was also used [34]. In this classification, the evaluation of abdominal pain is a criterion. We assumed that determining whether an infant has abdominal pain was difficult; therefore, we used the classification by Cotton et al. [33].

### 2.3. Inclusion and Exclusion Criteria

The articles were included for the study if they met the following criteria:

#### 2.3.1. Population

Studies on children diagnosed with common bile duct stone and underwent endoscopic sphincterotomy and/or endoscopic papillary balloon dilation were considered.

#### 2.3.2. Type of Study

Original articles on EST in more than 15 children were eligible for inclusion. Studies that did not indicate success rates of stone removal or complications with EST were excluded. All study designs of EPBD in children were eligible for inclusion. Furthermore, review articles or double publications were crosschecked and excluded to prevent duplication.

#### 2.3.3. Systematic Literature Search

The search algorithm for Medline (via PubMed) and Web of Science was structured by the combination of Medical Subject Headings terms, such as children, common bile duct stone, choledocholithiasis, endoscopic retrograde cholangiography, endoscopic sphincterotomy, and endoscopic papillary balloon dilation. The search period was between January 1995 and December 2023. Only English language publications were included. Furthermore, reference lists of selected articles were searched manually for potential additional studies.

#### 2.3.4. Outcomes

Success rate of stone removal, post-EST and -EPBD complications, grade of complications according to Cotton’s criteria [33], and mortality rate were the outcome parameters.

This study was approved by the Ethics Committee of Tokyo Women’s Medical University (approval number: 5728, 10 December 2021) in accordance with the Declaration of Helsinki of 1964 (revised in 2013).

## 3. Results

### 3.1. Cases of Endoscopic Papillary Balloon Dilation

#### 3.1.1. Patient 1

A 6-month-old girl presented with jaundice due to CBDS. She had undergone open heart surgery under cardiopulmonary bypass at the age of 5 months. Her body weight was 5 kg when she underwent EPBD. Her common bile duct was dilated because of impaction of stones up to 9.4 mm in diameter. The bile stones were removed twice via EPBD using a balloon catheter, and no complications were observed. She underwent laparoscopic cholecystectomy, following recovery from jaundice. The post-EPBD period was 6 years and 6 months, and bile stone recurrence and complications were not observed.

#### 3.1.2. Patient 2

**Figure 1:**
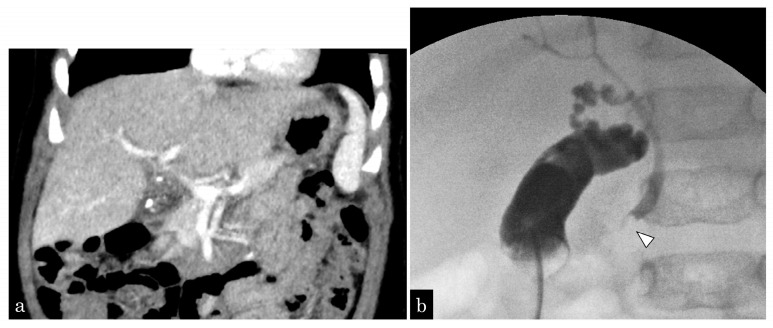
A 5-month-old girl presented with vomiting and revealed gallbladder and common bile duct stones on ultrasonography. (**a**) Enhanced computed tomography (CT) showing multiple small gallstones and gallbladder swelling. (**b**) The patient developed septic shock due to cholangitis. Emergency gallbladder drainage was performed, and a bile stone impacting the distal common bile duct (white arrowhead) was noted. A 4-Fr ENBD tube was inserted.

**Figure 2:**
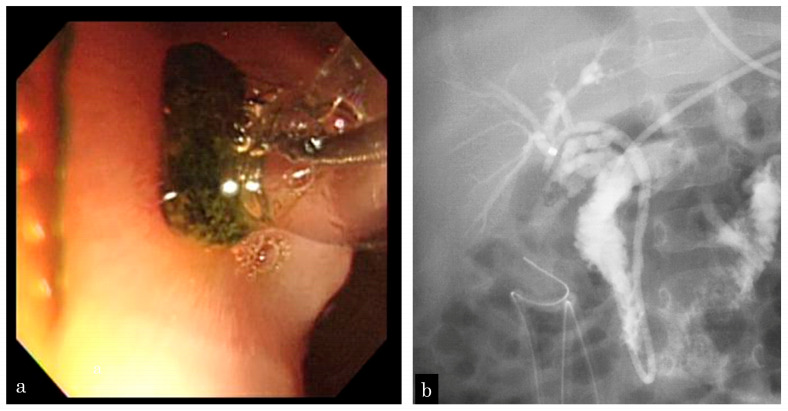
A 5-month-old girl who presented with vomiting and revealed gallbladder and common bile duct stones on ultrasonography. (**a**) EPBD is performed following recovery from cholecystitis and sepsis, and the stone is removed by one balloon pulling. (**b**) Following EPBD, a 5-Fr prophylactic ENBD tube is inserted, and no residual stone is revealed by cholangiography.

A 5-month-old girl presented with vomiting. Ultrasonography (US) revealed GBSs and gallbladder wall thickness. She presented with sepsis and urgently underwent gallbladder drainage (Figure 1a). During surgery, one CBDS impacting the distal common duct was observed, and an ENBD tube was inserted (Figure 1b). After recovery from cholecystitis and sepsis, EPBD was performed for CBDSs (Figure 2a,b). As the stone was 3 mm in size, the papilla was dilated by 3 mm in 30 s, and the stone was removed by one balloon pulling. The next day, after EPBD, serum lipase (normal range: 13–49 IU/L) levels increased to 1695 IU/L and returned to normal on the fifth day. After pancreatis improved, she underwent a cholecystectomy. The post-EPBD period was 4 months, and her clinical course was uneventful.

#### 3.1.3. Patient 3

**Figure 3:**
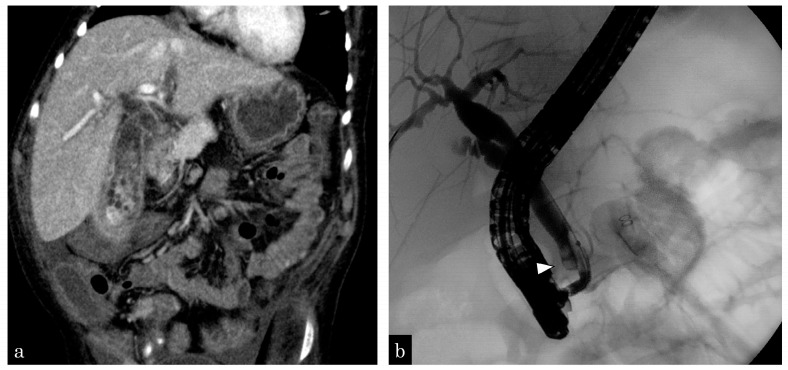
An 8-year-old boy complained of gallbladder and common bile duct stones. (**a**) Enhanced computed tomography (CT) revealing multiple small gallstones and gallbladder swelling. (**b**) The patient has developed septic shock due to cholangitis. Emergency gallbladder drainage is performed, and a bile stone impacting the distal common bile duct (white arrowhead) is observed. A 4-Fr ENBD tube is inserted.

An 8-year-old boy with trisomy 13 complained of jaundice and abdominal pain. Computed tomography revealed gallbladder wall swelling and multiple stones in the gallbladder and CBD (Figure 3a). Percutaneous transhepatic gallbladder drainage and ENBD tube insertion were performed for cholangitis and bile stones. After the improvement of cholecystitis, the patient simultaneously underwent laparoscopic cholecystectomy and EPBD. During surgery, 55 small bile stones in the gallbladder and 2 stones in the CBD were observed (Figure 3b). All bile stones were removed twice via EPBD, using a balloon catheter. One day after EPBD, lipase levels increased to 4999 IU/L but returned to normal on the second day. Five years have passed since the surgery and EPBD and no complications have been observed.

#### 3.1.4. Patient 4

A 7-year-old boy complained of abdominal pain and vomiting. Blood examination showed pancreatic enzyme level elevation, and US revealed pancreatic swelling. Magnetic resonance cholangiopancreatography showed no abnormality in the pancreaticobiliary system. ERCP showed a relatively long common channel, with one stone seen in the lower part of CBD. The stone was successfully removed via EPBD. One day after EBPD, serum lipase levels increased to 1923 IU/L and returned to normal on the third day. After the improvement pancreatitis, he underwent hepaticojejunostomy. Post-EPBD period was 9 months and no complication related to EPBD was observed.

All cases of CBD removal by EPBD were completed without complications of bleeding or perforation. Post-EPBD follow up ranged from 4 to 75 (mean 35) months, with no EPBD-associated complications.

### 3.2. A Review of Children Treated via EST

Studies on children treated via EST in high-volume centers since 2004 are presented in Table 2 [11,12,13,14,15,16,17,18]. The success rate of stone removal was high, ranging from 83% to 100% (mean 96%). The PEP incidence by EST at high-volume centers of pediatric facilities is described by dividing the number of PEP cases by the total number of ERCPs [12,13,16,17]. In this study, the percentages were calculated by dividing the number of PEP cases by the number of EST cases performed. This method of calculation is considered to be a more realistic estimate of PEP incidence in children, with rates ranging from 1.3% to 5.4% (mean 4.4%). The frequency of bleeding following EST in children ranged from 1.3% to 5.4% (mean 2.7%) and was calculated in a similar way to PEP.

### 3.3. A Review of Children Treated by EPBD

Studies on children treated by EPBD are shown in Table 3. Only a few studies on EPBD in children aged <15 years have been conducted [29,30,31]. Comorbidities were noted in in 5 of 13 children. The mainly used balloon diameter was 6–8 mm and balloon duration time was 15–75 s. Although the number of EPBD cases in children is small, the success rate of stone removal was 100%. In children, all EPBD-related complications were PEP, with a frequency of 6 of the 13 cases (46%) (5 mild cases and 1 moderate case).

## 4. Discussion

In our series, all four children with CBDS, including two infants, were successfully treated by EPBD without complications of bleeding or perforation. Although three of the four children developed PEP (mild, 1; moderate, 2) following EPBD, serum lipase levels in all cases returned to normal on day 3. The clinical course of post-EPBD was uneventful, with no EPBD-associated complications.

Several studies have attributed the lower incidence of long-term stone recurrence and cholangitis in the EPBD group compared with that in the EST group to papillary function preservation [23,24,25]. In contrast, the risk of PEP has been reported to be higher than that of EST [23,24,25,35]. Therefore, the European Society of Gastrointestinal Endoscopy (ESGE) clinical guidelines for CDBSs state that EPBD without EST is primarily indicated for patients with abnormalities in coagulation and stones smaller than 8 mm [36]. The PEP incidence by EST at high-volume centers of pediatric facilities [11,12,13,14,15,16,17,18] ranges from 1.3% to 5.4% (mean 4.4%). According to a meta-analysis of adults, the frequency of PEP ranged from 0% to 18.8% [24,25], suggesting that PEP frequency is not much different from that in children. The frequency of bleeding following EST ranged from 1.3% to 5.4% (mean 2.7%) in children vs. 0–26% (mean 3.4%) in adult [24,25], and the frequency of occurrence was not expected to differ from that in children. In adult cases, the grade of bleeding after EST was reported variously as moderate (45.8–71%) or severe (25–29%) [37,38]. Anticoagulants, cirrhosis, and end-stage renal disease were noted as risk factors for bleeding [38]. However, the grade of post-EST bleeding was mostly mild (87%) in average risk adult patients [39]. Generally, the risk factors of post-EST bleeding in children are rare; seven cases of bleeding were mild (64%) and four cases were moderate (36%), indicating a high rate of moderate bleeding. In children, bleeding following EST is associated with a high risk of blood transfusion. Conversely, in EPBD in children [29,30,31], all complications were PEP, with a frequency of 6 of the 13 cases (46%) (5 mild cases and 1 moderate case), which was clearly higher than that for EST. This PEP frequency in children is also higher than that in adults who underwent EPBD [0–11.3% (mean 7.0%)] [24,25]. Balloon diameter and dilation time have been identified as factors associated with PEP following EPBD [36]. Regarding balloon diameter, a comparison has been made between EPBD and endoscopic papillary large balloon dilation (EPLBD) in adults [40]. The frequency of PEP is lower with EPLBD than with EPBD [41,42]. Studies [30,31] on children have mentioned the use of a balloon diameter (4–8 mm) slightly smaller than the common bile duct diameter (5–10 mm) to prevent PEP. In our series, we selected a balloon diameter (3–4 mm) with the same size as CBDSs. Therefore, the balloon diameter in our series (3–4 mm) was smaller than that in other studies [29,30]. Even with the use of a small-diameter balloon, the situation in children may be similar to that in which EPLBD is performed. However, PEP in children was more frequent than EPLDB in adults. The ESGE [36] and the American Society for Gastrointestinal Endoscopy [43] also state that a dilation time longer than 2 min carries a lower risk of PEP. In contrast, Wang et al. (2021) [44] presented a systematic review and meta-analysis of EPBD according to balloon dilation time. They classified the dilation times into short dilation times of <1 min and long dilation times of >1 min and noted no difference in complications or successful stone removal rates between the short and long dilation times. In children who underwent EPBD [29,30,31], the dilation times were relatively short, ranging from 15 to 75 s. This is believed to be because children have softer tissues than adults, which may have led to the shorter balloon dilation time. Furthermore, we set a short dilation time (30 s) for infants. PEP frequently occurred in children who underwent EPBD, mostly with mild cases, with a rapid increase in pancreatic enzyme levels following EPBD; however, most children had a rapid return to normal range within a few days. No mortality or severe pediatric cases of PEP due to EPBD were reported. Therefore, we supposed that papillary edema rapidly improves following EPBD, owing to soft tissues in children. Based on the correlation between common bile duct diameter and post-EPBD pancreatitis occurrence, there is a lack of systematic reviews and meta-analyses in adults. In our pediatric series and other related studies where common bile duct diameter is described [30], pancreatitis was observed following EPBD in six children with common bile duct diameters ranging 3.5–10 mm (mean 6.3 mm), but not in three children with diameters ranging 6–9.4 mm (mean 7.4 mm). No evident association was found between common bile duct diameter and post-EPBD pancreatitis.

This study had some limitations. The most significant of these was the small number of pediatric EPBD cases, particularly in infants. The guidelines of the ESGE [36] and the American Society for Gastrointestinal Endoscopy [43] have recommended EPBD in adults and not in children. More reports on pediatric EPBD cases are required. Subsequently, further investigation into the association between the incidence of PEP and the common bile duct diameter, balloon diameter, and dilation time in children is needed. In particular, the balloon dilation time for pediatric EPBD was relatively short, and it will be necessary to perform dilatations of 2 min or longer to compare cases for future studies. Also, we hope to report long-term post-EPBD outcomes for children in the future.

## 5. Conclusions

Stone removal via EBPD in children is expected to have a high success rate, with a very low risk of bleeding and perforation. The frequency of PEP is high, with most cases being mild; the post-treatment course is uneventful when post-EBPD management is correctly performed. In children who require a long clinical follow up after CBDS removal, papillary function preservation is expected to prevent stone recurrence and biliary tract infection and is considered an effective method.

## Figures and Tables

**Table 1:** Features of pediatric patients with common bile duct stones treated with endoscopic papillary balloon dilatation.

Patients	Sex	Age	Comorbidities	CBD	CBDSs	
				Diameter (mm)	Number	Size (mm)
1	F	6 mos	Post cardiac surgery	9.4	2	4
2	F	5 mos	None	3.5	1	3
3	M	8 yrs	13 trisomy	8.5	2	4
4	M	7 yrs	PBMJ	4	1	4

Abbreviations: mos, months old; yrs, years old; M, male; F, female; CBD, common bile duct; CBDSs, common bile duct stones; PBMJ, pancreaticobiliary maljunction.

**Table 2:** Studies on endoscopic retrograde cholangiopancreatography and endoscopic sphincterotomy in children with common bile duct stones in a high-volume center.

Author and Year	Total Number of Patients/ERCPs	Number of Patients Who Underwent EST	Success Rate of Stone Removal by EST, % (Success/Fail)	EST Complications, n (%)
Rocca R	38/44	37	100% (14/0)	Bleeding 2 (5.4%)
2004 [11]				(mild 2)
Cheng CL	245/329	135	96% (23/1)	Bleeding 5 (3.7%)
2005 [12]				(mild 5)
				PEP 13 (9.6%)
(NC)				
Issa H	125/NC	62	97% (34/1)	Bleeding 1 (1.6%)
2007 [13]				(moderate 1)
				PEP 4 (6.4%)
				(mild 4)
Jang JY	122/245	150	NC	Bleeding 2 (1.3%)
2010 [14]				(moderate 2)
Otto AK	167/231	96	98% (54/1)	Bleeding 2 (2%)
2011 [15]				(mild 1, moderate 1)
				PEP NC
Trendle DM	111/154	65	98% (64/1)	Bleeding 1 (1.5%)
2013 [16]				(moderate 1)
				PEP 3 (4.6%)
				(mild 3)
				Other 1 (1.5%)
				(moderate 1)
Fishman DS	44/NC	44	NC	PEP 2 (4.5%)
2016 [17]				(mild 2)
Keane MG	90/111	18	83% (15/3)	NC
2018 [18]				
Total		545		

Abbreviations: ERCP, endoscopic retrograde cholangiopancreatography; EST, endoscopic sphincterotomy; PEP, post-endoscopic retrograde cholangiopancreatography pancreatitis; NC, no contributions.

**Table 3:** Endoscopic papillary balloon dilation in children under 15 years old with common bile duct stones.

Author and Year	Age/Sex	Comorbidities	Balloon Size, mm	Dilation Time	Stone Removal	Post-EPBD Complications
Tarnasky PR	15 yrs/F	None	6 to 8	NC	Yes	None
1998 [29]	12 yrs/F	SCA	6 to 8	NC	Yes	None
	4 yrs/M	None	6 to 8	NC	Yes	None
Osanai M	11 yrs/F	HS	6	15 s	Yes	Mild PEP
2011 [30]	7 yrs/F	None	6	15 s	Yes	Mild PEP
	10 yrs/F	None	6	15 s	Yes	Mild PEP
	9 yrs/F	None	4	15 s	Yes	None
	13 yrs/M	None	6	15 s	Yes	None
Sogo T	7 yrs/F	None	8	75 s	Yes	None
2013 [31]						
Kouchi	6 mos/F	Post cardiac	4	60 s	Yes	None
2024		surgery				
	5 mos/F	None	3	30 s	Yes	Moderate PEP
	8 yrs/M	13 trisomy	4	30 s	Yes	Mild PEP
	7 yrs/M	PBMJ	4	60 s	Yes	Mild PEP

Abbreviations: mos, months old; yrs, years old; M, male; F, female; SCA, sickle cell anemia; HS, hereditary spherocytosis; NC, no contributions; EPBD, endoscopic biliary balloon dilation; PEP, post-endoscopic retrograde cholangiopancreatography pancreatitis.

## Data Availability

Data generated or analyzed during this study are available from the corresponding author by request subject to institutional review and a data use agreement.
